# Deciphering the nexus between long non-coding RNAs and endoplasmic reticulum stress in hepatocellular carcinoma: biomarker discovery and therapeutic horizons

**DOI:** 10.1038/s41420-024-02200-2

**Published:** 2024-10-24

**Authors:** Himanshi Goyal, Sachin Parwani, Jyotdeep Kaur

**Affiliations:** Department of Biochemistry, Postgraduate Institute of Medical Education and Research, Chandigarh, India

**Keywords:** Long non-coding RNAs, Epigenetics

## Abstract

Hepatocellular carcinoma (HCC) remains a significant global health challenge with few effective treatment options. The dysregulation of endoplasmic reticulum (ER) stress responses has emerged as a pivotal factor in HCC progression and therapy resistance. Long non-coding RNAs (lncRNAs) play a crucial role as key epigenetic modifiers in this process. Recent research has explored how lncRNAs influence ER stress which in turn affects lncRNAs activity in HCC. We systematically analyze the current literature to highlight the regulatory roles of lncRNAs in modulating ER stress and vice versa in HCC. Our scrutinization highlights how dysregulated lncRNAs contribute to various facets of HCC, including apoptosis resistance, enhanced proliferation, invasion, and metastasis, all driven by ER stress. Moreover, we delve into the emerging paradigm of the lncRNA-miRNA-mRNA axis, elucidating it as the promising avenue for developing novel biomarkers and paving the way for more personalized treatment options in HCC. Nevertheless, we acknowledge the challenges and future directions in translating these insights into clinical practice. In conclusion, our review provides insights into the complex regulatory mechanisms governing ER stress modulation by lncRNAs in HCC.

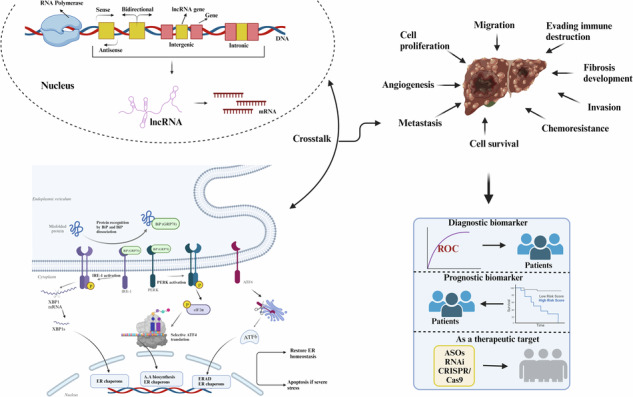

## Facts

- Hepatocellular carcinoma is the second leading cause of cancer-related mortality, emphasizing the urgent need for innovative therapeutic approaches.
- Increased demand for protein synthesis in tumor cells activates the UPR pathway as an adaptive mechanism.
- The intensity and duration of UPR dictates the cells’ pro-survival and pro-apoptotic fate.
- LncRNAs exert their molecular function through RNA-protein, RNA-RNA, and RNA-DNA interactions and via miRNA sponging.
- Investigating the complex interplay between the UPR pathway and lncRNA holds the promise for the development of novel diagnostic tools and therapeutic interventions in HCC.

## Open questions

1. How can the crosstalk between UPR-related genes and lncRNAs be leveraged to develop novel therapeutic interventions for HCC and other cancers?
2. What strategies can be employed to overcome the limitations of current investigations and accelerate the translation of lncRNA-based therapies into clinical practice for HCC and other malignancies?
3. How can we optimize the utilization of lncRNAs as molecular markers to predict therapeutic responses and guide treatment decisions in HCC patients?
4. What are the potential ethical considerations and challenges associated with the targeted modulation of lncRNAs and ER stress pathways in cancer therapy?
5. How do lncRNAs influence the sensitivity of HCC to conventional treatments such as radiotherapy and chemotherapy, how can this be harnessed to enhance treatment efficacy?

## Introduction

Hepatocellular carcinoma (HCC) stands as a significant global health concern, holding the fifth position among the most prevalent human malignant tumors [1]. HCC, the predominant form of primary liver cancer stands as the second leading cause of cancer-related mortality globally, and is characterized by poor prognosis [2]. Thus, there is an urgent need to innovate and develop new therapeutic interventions [3]. Tumor cells exhibit an increased demand for the synthesis of proteins leading to an endoplasmic reticulum (ER) stress [4], which further activates the unfolded protein response (UPR) to maintain ER homeostasis.

ER is one of the largest subcellular compartments, which plays a pivotal role in many biological processes like co-translational protein folding in the cytosol, Ca2+ homeostasis, vesicular transport, and the biosynthesis of steroids and lipids. There is a fine-tuning of the ER activities by the chaperons, proteolytic enzymes, and Ca2+ transporters and channels. Disruptions to ER homeostasis leading to ER stress can occur due to both physiological and pathological triggers, nutrient deprivation, oxidative stress, hypoxia, and mutations in ER-related genes. In order to overcome the ER stress, cells have developed an ER-nucleus signaling pathway known as the unfolded protein response (UPR), which either resolves ER stress via a pro-adaptive mechanism or initiates cell death via apoptosis. Under the conditions of mild to moderate ER stress, the UPR has the tendency to eliminate unfolded or misfolded proteins for restoration of ER homeostasis, known as the ‘adaptive’ UPR. However, under prolonged ER stress, the UPR triggers intrinsic apoptotic pathways as the result of excessive activation, termed as ‘maladaptive’ UPR.

### Mechanism of UPR activation

**Fig. 1:**
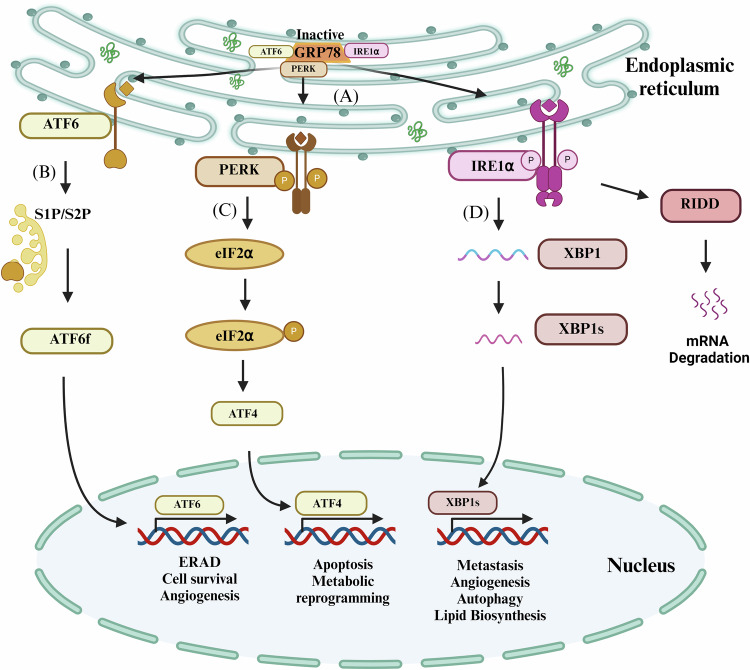
Various stress at ER levels causes the induction of UPR pathway. UPR: A signaling pathway in response to ER stress, illustrating the three primary signaling branches of the UPR activated by ER stress sensors: ATF6, PERK, and IRE1α (**A**) Activated GRP-78 causes the release of three ER stress sensors: ATF-6, PERK, and IRE-1α, (**B**) ATF6 gets fragmented as the result of GRP-78 activation generating ATF6f which gets translocated to the nucleus to activate ERAD pathway, (**C**) phosphorylated PERK causes phosphorylation of eIF-2α, which inhibits overall translation but activates ATF4 inducing apoptosis, (**D**) Phosphorylation of IRE1α generates the spliced product of XBP1 i.e. XBP1s, affecting autophagy, metastasis and lipid biosynthesis. (Created with Biorender. com).

Nutrient unavailability, hypoxia, low pH, accumulation of reactive oxygen species (ROS), and genetic alterations in cancer cells can trigger ER stress in cancer. This involves the accumulation of the misfolded proteins in ER thereby activating UPR via three ER membrane sensors i.e,. inositol requiring enzyme 1alpha (IRE-1α), activating transcription factor-6 (ATF-6) and protein kinase R-like ER kinase (PERK) [5]. Under normal conditions, these sensor proteins remain in their inactive state due to their binding with the glucose-regulated protein 78 (GRP78) [6]. However, under increased ER stress, association with GRP78 is disrupted which commences the UPR signal transduction. The intensity and duration of UPR determines the pro-survival or pro-apoptotic state of the cell [7]. Moderate ER stress can induce cell proliferation, angiogenesis, chemoresistance, and metastasis, whereas extreme ER stress can cause cell death [8] (Fig. 1). Khaleed et al. 2022 reviewed the mechanism by which HCC cells adopt the drug resistance phenotype via various resistance mechanisms [9]. Growing evidence also suggests that intense ER stress and maladaptive UPR play a significant role in the development of hepatic diseases {reviewed by Ajoolabady et. al. [10]}.

**Fig. 2:**
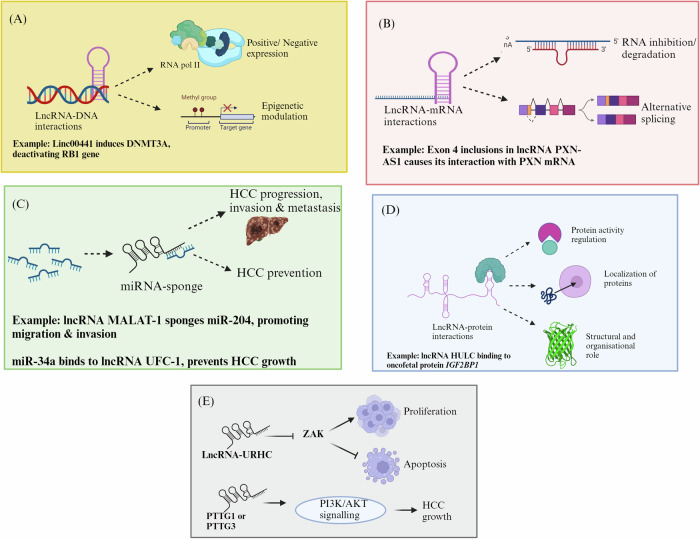
LncRNAs regulate the gene expression by various mechanisms including epigenetic silencing, splicing, miRNA sponging, interacting with proteins and other mechanisms. Functional mechanisms of lncRNAs: (**A**) Epigenetic silencing-lncRNA interacting with DNA regulates RNA pol II positively or negatively and modulates epigenetics via DNA methylase activity and histone modifications (**B**) splicing modulations- lncRNA can bind to mRNA further causing their inhibition or degradation and also affect the alternative splicing of mRNAs (**C**) miRNA sponging- lncRNAs bind to the miRNAs and cause their sponging to either activate or inhibit the target gene, affecting HCC progression (**D**) lncRNA-protein interactions- lncRNAs directly binds to the translational unit, affecting their activity, localization or their structure (**E**) Other mechanisms. (Created with Biorender. com).

Cancer cells respond to ER stress by altering the expression of different non-coding RNAs, including microRNA (miRNA), long noncoding RNA (lncRNA), and circulatory RNA (circRNAs) [11]. ncRNAs include lncRNAs with lengths greater than 200 nucleotides, miRNAs with length 19-24 nucleotides, and circRNAs that are single-stranded. Most of the lncRNAs are transcribed in the RNA polymerase II-dependent transcription, which then undergoes capping at 5’end, polyadenylation at 3’ end, and splicing, earning them the description of ‘mRNA-like’ [12]. However, some other lncRNAs that originated from Pol I, Pol III, or the precursors like introns and repetitive elements lack the 7- methyl guanosine caps as well as the poly- A tails, which are referred to as ‘transcripts of unknown function’ [13]. In comparison to the protein-coding genes, lncRNAs can also be intergenic, antisense, or intronic. In addition to this, lncRNAs can also arise from pseudogenes, some of which are functional [14]. LncRNAs exert their regulatory functions through diverse mechanisms, which include the regulation at replication, transcription, and translational levels. LncRNAs serve as the structural scaffolds for nuclear or cytoplasmic complexes and modulate gene expression [15]. LncRNAs also exert their molecular functions through RNA-protein interactions, RNA-RNA interactions, and RNA-DNA interactions (Fig. 2). Dysregulation of lncRNAs has been incriminated by its regulation of the UPR pathway in the progression of different types of cancer [16].

This review emphasizes the modulation of the ER stress by the lncRNAs as the epigenetic modifiers in HCC and focuses on how the lncRNA-miRNA-mRNA axis can be addressed in the future to develop a novel diagnostic biomarker or a therapeutic intervention for HCC.

### Search strategy

**Fig. 3:**
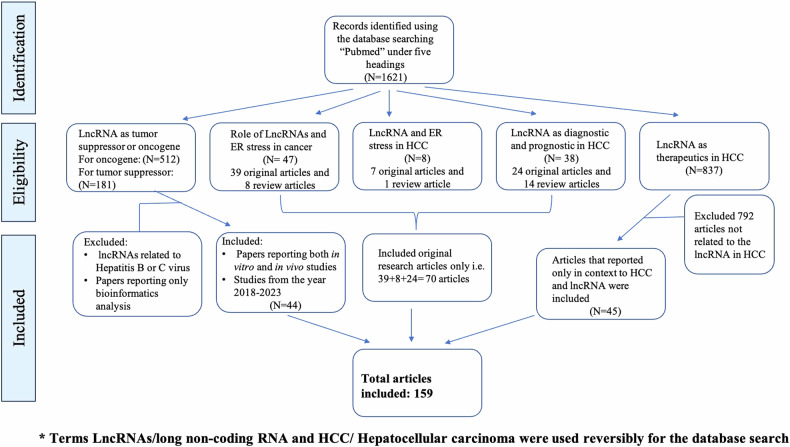
Search strategy followed for the screening of the articles that were to be included in the review article.

To select the articles for the review article, we performed a literature search using an electronic database, Pubmed. We tried to include the latest articles exclusively using the different terms, details of which are depicted in Fig. 3.

## LncRNAs as oncogenes in HCC

Certain lncRNAs promote the development and progression of cancer by various mechanisms, for example, inhibiting apoptosis, leading to enhanced cell survival by interacting with chromatin modifiers, by altering the DNA methylation or histone modifications, thereby promoting oncogene expression and repression of tumor suppressor gene. For instance, silencing lncRNA SLC7A11-AS1 effectively suppressed HCC progression, a finding corroborated by in vivo and in vitro experiments [17]. Additionally, it was demonstrated that METTL3 facilitated m6A modification of this lncRNA SLC7A11-AS1, which enhances its expression in HCC. Furthermore, SLC7A11-AS1 has been shown to downregulate KLF9 by influencing STIP-1 homology and U-box containing protein 1 (STUB1)-mediated ubiquitination degradation, allowing KLF9 to elevate PH domain and leucine-rich repeat protein phosphatase 2 (PHLPP2) expression, resulting in AKT pathway inactivation [17]. In HCC, a study identified an elevated level of the lncRNA HOMER3-AS1, which is associated with increased HCC growth, migration, and invasion and thus has poor patient survival. It has been implicated in the recruitment and polarization of M2 macrophages, further facilitating cancer cell proliferation [18]. LncRNAs have been suggested to act as competitive endogenous RNA (ceRNA) molecules that competitively interact with proteins and genes. However, it is still unclear how these molecules affect the development of HCC [19]. For example, the findings of Chen K et al. suggested that lncRNA SNHG6 operates as a ceRNA, competitively binding to miR-204-5p to increase E2F1 expression, and promoting the G1-S phase transition, which leads to HCC tumorigenesis [20]. LncRNA-miRNA network plays a pivotal role in cancer, acting as oncogenes that modulate gene expression and contribute to the malignancy of tumors. For instance, lncRNA CCAT2 preferentially inhibits miR-145 maturation, resulting in lower levels of mature miR-145 in HCC cells [21]. This inhibition has been shown to affect the progression of HCC. CCAT2 knockdown reduces HCC cell proliferation and metastasis, according to functional studies conducted in vitro and in vivo. emphasizing the role of miR-145 in CCAT2-mediated HCC growth [22]. The role of CCAT2 has also been reported previously in HCC progression via regulation of miR-4496/Atg5 axis [23]. The findings by Cheng D et al. reported the molecular interplay between two important ncRNAs in HCC i.e., HOTAIR and miR-122. HOTAIR exerts epigenetic regulation by decreasing miR-122 expression through DNMTs-induced DNA methylation, resulting in dysregulated Cyclin G1 expression in HCC cells [24]. Moreover, research has shown that elevated expression of HOTAIR is associated with sorafenib resistance in HCC cells [24, 25], A recently identified lncRNA, LL22NC03-N14H11.1 has been shown to interact with the Myb proto-oncogene (c-Myb) to reduce expression of leucine zipper-like transcription regulator 1 (LZTR1), hence decreasing the ubiquitination of H-RAS thereby, activating MAPK signalling pathway [26]. According to a recent study in HCC, the combination of E2F1 with super-enhancers (SEs) aided in the expression of LINC01004. Surprisingly, limiting SE activity resulted in a significant decrease in LINC01004 expression. These findings can potentially improve our understanding of the molecular mechanisms underlying hepatocarcinogenesis and the oncogenic role of lncRNAs [27]. Therefore, oncogenic lncRNAs in HCC play crucial roles in tumor development and progression through various mechanisms, including the inhibition of apoptosis, modulation of chromatin states, and alteration of DNA methylation and histone modifications. Table 1 outlines the comprehensive compilation of lncRNAs that act as oncogenes and are associated with the progression of HCC.

**Table 1:** Oncogenic lncRNAs and their Roles in HCC.

LncRNAs	Biological Function	Mechanistic Pathway	Reported in other Cancers	Reference
UBE2CP3	Angiogenesis, migration, and invasion	ERK/HIF-1α/p70S6K/VEGFA signaling	Gastric cancer [108]	[109]
LINC01132	Migration, invasion, and metastasis	NRF1/DPP4 signaling axis	Ovarian cancer [110]	[111]
NIHCOLE	DNA repair	NHEJ-mediated DSB repair	Not reported	[112]
RP11-386G11.10	Cellular proliferation, and metastasis	ZBTB7A/RP11-386G11.10/miR-345-3p/HNRNPU axis	Not reported	[113]
MALAT1	Invasion, migration, and angiogenesis	mTORC1–4EBP1 axis	Breast cancer [114], Prostate cancer [115], and Colon cancer [116]	[117]
HOXD-AS1	Migration, invasion, and metastasis	STAT3/miR-130a-3p, SOX4	Cervical cancer [118], and Pancreatic cancer [119]	[120]
Lnc-EGFR	T-reg cell differentiation, and immunosuppression	AP-1/NF-AT1 axis	Glioblastoma multiforme [121], and Tongue cancer [122]	[123]
lncRNA-PDPK2P	Migration, invasion, and metastasis	PDK1/AKT/caspase 3 signaling	Not reported	[124]
MAPKAPK5-AS1	Cellular proliferation, EMT, and metastasis	PLAGL2/EGFR/AKT Pathway	Non-small cell lung cancer [125]	[126]
KDM4A-AS1	Migration, invasion, and metastasis	miR-16-5p/ANXA11 signaling pathway	Not reported	[127]
TUG1	Cell survival, proliferation, migration, and invasion	TUG1/ hsa-miR-582-5p / Siglec-15 axis	Colorectal cancer [128], and Bladder cancer [129]	[130]
DLGAP1-AS1	Cellular proliferation	miR‐486‐5p/H3F3B axis	Glioblastoma [131]	[132]
MCM3AP-AS1	Cell survival, proliferation, and cell cycle progression	miR-194-5p/FOXA1 axis	Small cell lung cancer [133]	[134]
LASP1-AS	Migration, invasion, and metastasis	Not Known	Not reported	[135]
HOXA11-AS	Cell proliferation, and cell cycle progression	HOXA11-AS /EZH2/DUSP5 axis	Glioblastoma [136], and Breast cancer [137]	[138]
LEF1-AS1	Cell cycle progression	miR‐148a‐lnc‐UCID‐DHX9‐CDK6 axis	Osteosarcoma [139]	[140]
ZFAS1	Invasion, and metastasis	miR-150/ ZEB1, MMP14, and MMP16 axis	Colorectal cancer [141], and Pancreatic cancer [142]	[143]
OR3A4	Migration, invasion, and angiogenesis	AGGF1/AKT/mTOR pathway	Ovarian cancer [144], and Osteosarcoma [46]	[145]
MYCNOS	Migration, invasion, and EMT	Not Known	Glioblastoma [146]	[147]
MEG8	Cell proliferation, migration, invasion, and EMT	miR-367-3p/14-3-3ζ/TGFβR1 axis	Non-small cell lung cancer [148]	[149]
LINC00657	Cell proliferation, migration, and invasion	miR-424/PD-L1 axis	Cervical cancer [150]	[151]
linc00467	Cell cycle progression, and migration	MiR-18a-5p/NEDD9 axis	Prostate cancer [152]	[153]
HAGLROS	Cell proliferation, and metastasis	HAGLROS/miR-26b-5p/KPNA2 axis	Ovarian cancer [154], and Esophageal cancer [155]	[156]
BBOX1-AS1	Drug resistance, cell proliferation, and migration	miR-361-3P/PHF8 axis	Colorectal cancer [157]	[153]

## LncRNAs as tumor suppressors in HCC

Many studies have suggested the role of various lncRNAs in governing different cancer hallmarks such as migration, invasion, angiogenesis, and progression of HCC. For instance, low expression of lncRNA LINC01146 is linked with the clinical manifestations of malignancy, correlating with the unfavourable prognosis of HCC. Also, the in vivo studies showed that elevating LINC01146 notably suppresses tumor growth [28]. Wang F et al. underscore the capacity of lncRNA CADM1-AS1 to hinder HCC proliferation via AKT/GSK-3β signaling pathway, thereby orchestrating alterations in key cell cycle regulators to prevent the transition from G0/G1 to S phase transition, both in culture as well as mouse model of HCC [29]. The concise list of various lncRNAs acting as tumor suppressor, along with their biological functions, is comprehensively described in Table 2.

**Table 2:** Tumor suppressor lncRNAs and their roles in HCC.

LncRNA	Biological Function	Mechanistic Pathway	Reported in other Cancers	Reference
Linc01612	Apoptosis induction	miR-494/ATF3/p53 axis	Not reported	[158]
Linc-USP16	Suppresses proliferation, invasion, and metastasis	miR-21/miR-590-5p/PTEN axis	Not reported	[159]
LOC107985656	Growth inhibition	miR-106b-5p/LATS1 axis	Not reported	[160]
LINC01554	Proliferation, and invasion	PI3K/Akt/mTOR signaling pathway	Non-small cell lung cancer [161]	[162]
FENDRR	Inhibition of Treg-mediated immune escape, and apoptosis induction	GADD45B/miR-423-5p axis	Gastric cancer [163], and Cervical cancer [164]	[165]
TPTEP1	Suppresses proliferation, invasion, and metastasis	miR-454-3p/DLG5 axis	Gastric cancer [166], and Non-small cell lung cancer [167]	[168]
LIMT	Suppressed proliferation, invasion, and metastasis	EGF/EGFR signaling pathway	Breast cancer [169]	[170]
LINC00472	Inhibition of invasion, and migration	miR-93-5p/PDCD4 pathway	Lung adenocarcinoma [171], and Colorectal cancer [172]	[173]
LINC02428	Suppressed proliferation, invasion, and metastasis	KDM5B/IGF2BP1 feedback loop	Not reported	[174]
FAM99A	Suppressed proliferation, invasion, and metastasis	Not known	Not reported	[175]
LINC02499	Suppressed proliferation, and invasion	Not known	Not reported	[176]
LINC01488	Cell cycle arrest, inhibition of EMT, and metastasis	miR-124-3p/miR-138-5p/vimentin axis	Not reported	[177]
LncRNA-CASC2c	Suppressed proliferation, invasion, and migration	ERK1/2 and Wnt/β-catenin signaling pathway	Non-small cell lung cancer [178]	[179]
LPAL2	Suppressed proliferation, invasion, and migration	LPAL2/MMP9 axis	Not reported	[180]
WFDC21P	Suppress proliferation, and impedes metastasis	Nur77-WFDC21P-PFKP/PKM2 axis	Not reported	[181]
NONHSAT024276	Induce cell apoptosis, cell cycle arrest, invasion, and migration	NONHSAT024276/PTBP1 feedback loop	Not reported	[182]
DHRS4-AS1	Apoptosis induction	DHRS4-AS1/miR-522-3p/SOCS5	Non-small cell lung cancer [183]	[184]
MAGI2-AS3	Suppression of proliferation, invasion, and migration	MAGI2-AS3/miR-374b-5p/SMG1	Prostate cancer [185], and Breast cancer [186]	[187]
MEG3	Apoptosis induction	miR-9-5p/SOX11 axis	Prostate cancer [188], and Laryngeal cancer [189]	[91]
TP53TG1	Drug resistance	ERK signaling pathway	Gastric cancer [190], and Cervical cancer [191]	[192]

Invasion and migration are crucial processes that underpin the cancer metastasis and various lncRNAs are proven to inhibit these processes. In this context, lncRNA GAS5 has been reported to be altered in many cancers, including HCC. miR-135b directly binds to and inhibits the activity of GAS5. GAS5 normally acts to increase the production of RECK. Interestingly, miR-135b also targets and inhibits RECK. This regulatory cascade ultimately dampens MMP-2 expression, collectively contributing to the suppression of invasion processes [30]. In addition to this, the tumor-suppressing activity of GAS5 has been reported in HCC, where it acts as a negative modulator of miR-21 and various proteins involved in cancer cell invasion and migration [31]. Certain tumor suppressor lncRNAs exert their function through epigenetic mechanisms. For example, lncRNA MAGI2-AS3-protein complex is subsequently recruited to the RACGAP1 promoter region leading to decreased RACGAP1 expression via H3K4me2 demethylation in HCC cells [32]. On the other hand, there are other lncRNAs that modulate gene expression by interfering with autophagy and cellular pathways.For example, Xu et al. showed that increased WWOX-AS1 expression was related to decreased cell proliferation, migration, EMT, and increased cell death [33]. lncRNA NBR2 suppresses cytoprotective autophagy, thus reducing HCC cell growth. NBR2 has been found to inhibit Beclin 1-dependent autophagy by influencing the ERK and JNK pathways [34].

**Fig. 4:**
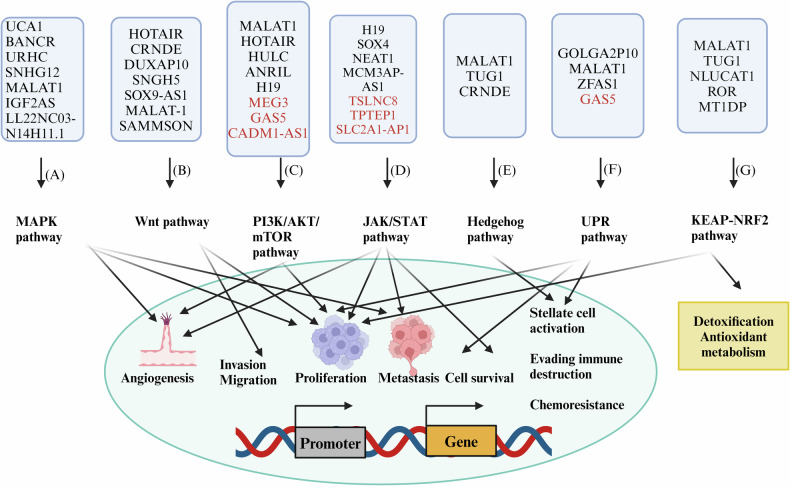
Diverse lncRNA-medicated signaling pathways targeting different hallmarks of cancer (lncRNAs written in black and red color represent oncogenes and tumor suppressors, respectively). **A** MAPK pathway upregulated by oncogenic lncRNAs further activates angiogenesis, metastasis and causes increased proliferation of cancer cells. **B** Wnt signaling pathway activated as the result of upregulation of oncogenic lncRNAs increases invasion and migration. **C** PI3K/AKT/mTOR pathway causes angiogenesis and increased proliferation in tumor cells, (**D**) Oncogenic and tumor suppressing lncRNAs affect JAK/STAT signaling pathway to induce metastasis, angiogenesis and cell proliferation. **E** Oncogenic lncRNAs activates the stellate cells via Hedgehog pathway. **F** UPR pathway activated by different lncRNAs induces stellate cell activation and cell proliferation (**G**) Oncogenic lncRNAs activates KEAP-NRF2 pathway which causes detoxification as well as cell proliferation. (Created with Biorender. com).

Hence, lncRNAs have emerged as critical regulators in a wide range of cancers, including HCC. However, the specific actions and underlying mechanisms of lncRNAs in HCC warrant further exploration. Figure 4 depicts an overview of the diverse signaling pathways by which lncRNAs exert their regulatory influence, ultimately modulating various hallmarks of cancer.

## Role of lncRNAs in ER stress: implications for cancer pathogenesis

LncRNAs can act as oncogenes promoting proliferation, invasion, and metastasis or as tumor suppressors inhibiting tumorigenesis. Many studies in the literature have thrown light on how lncRNAs act as regulators of the many genes and cellular pathways involved in various diseases and cancers, including HCC [16]. Consequently, there has been a rising interest in investigating the impact of dysregulated lncRNAs in cancer pathogenesis. However, their interactions with DNA, RNA, and proteins are intricate and context-dependent, necessitating advanced technologies and methodologies for comprehensive analysis. Increasing evidence suggests that ER stress is a double-edged sword during HCC development and progression. During ER stress, cancer cells either survive by inducing adaptive mechanisms or undergo cell death by apoptosis. The study of lncRNAs is relatively new in comparison to other genetic elements like mRNAs and miRNAs. As a result, research into their roles in specific cellular processes, including ER stress, is still in its early stages. Many lncRNAs remain functionally unannotated, with their precise roles and mechanisms of action yet to be determined. This lack of functional information hinders the ability to fully understand their involvement in ER stress. ER stress itself is a complex cellular response involving multiple signaling pathways and regulatory mechanisms. Disentangling the specific contributions of lncRNAs within this multifaceted process requires detailed and focused studies. The exploration of lncRNA mechanisms in ER stress remains a challenging yet promising area of research that warrants further attention. However, some of the studies have tried to elucidate the mechanism of ER stress-related lncRNAs in the tumor progression. Evidence has suggested that lncRNA GAS5 stimulates the UPR by binding to GRP78 and activates the caspase-9 and CHOP (C/EBP homologous protein) signaling pathways, which further activate caspase-3 and cause cell apoptosis in HepG2 cells [35]. Another lncRNA, MEG3, has been explored in the context of UPR in various cancers. A study showed that elevated levels of lncRNA MEG3 correlate with the increased expression of proteins related to ER stress pathways such as GRP78, PERK, IRE1α, and ATF6, and resulted in enhanced cytosolic to nuclear translocation of NF-κB in breast cancer cells and induced apoptosis in various cancer cells [36–38]. Apart from MEG3, MALAT-1 has also been extensively studied in different cancers, highlighting its role in tumor development and progression. A study indicates that thapsigargin (ER stress inducer) at low doses may enhance the migration of colorectal cancer cells by increasing the expression of the lncRNA MALAT1. The IRE1α/XBP1 and PERK/eIF2/ATF4 signaling pathways were both activated in conjunction with this rise in MALAT1 expression. However, the interaction between MALAT1 and XBP1 or ATF4 has not been explicitly validated to date [39]. The lncRNA LUCRC is substantially expressed in colorectal tumors and is essential for the growth, migration, and invasion of colorectal cancer cells in cultured cells as well as in xenografts. In the ER, LUCRC was discovered to control the expression of binding immunoglobulin protein (BIP) and other target genes of the UPR. In the blood plasma of individuals with colorectal malignancies, the presence of LUCRC emphasizes its clinical importance as a therapeutic target [40]. Recently, Zhang H et al. have underscored that SNHG1, a lncRNA regulated by Kruppel-like factor (KLF4), operates to dampen ER stress-induced apoptosis and propel the development of gliomas by orchestrating the elevation of inhibitor of apoptosis (IAP) family protein baculoviral IAP repeat containing 3(BIRC3) [41]. In addition to the role of ER stress associated lncRNAs in promoting migration, invasion, and inducing apoptosis, they are known to regulate the resistance to chemotherapy. Findings of Yao X et al. demonstrated that in MCF-7 cells, 5-flourouracil (5-FU)-induced ER stress enhanced the expression of GRP78 through the upregulation of octamer binding transcription factor 4 (OCT4), which may positively influence the expression of lncRNA MIAT, and thus contributing in resistance to 5-FU [42]. A recent study showed that altering lncRNA H19 can increase the anti-cancer effects of resveratrol by affecting cancer cell growth and migration, possibly through regulating ER stress-related pathways. These results advance knowledge of the molecular mechanisms underlying the connection between lncRNAs, organic substances like resveratrol, and cancer cell behavior. According to the research, targeting lncRNA H19 and ER stress pathways may be promising areas for developing new cancer treatment therapeutics [43]. The findings by Ding et al. indicate that decreased lncRNA CAS2 in non-small cell lung carcinoma (NSCLC) stabilizes PERK mRNA. This, in turn, activates the PERK/eIF2α/CHOP pathway of ER stress, leading to increased radiosensitivity or apoptosis in NSCLC cells exposed to irradiation [44]. Additionally, numerous experiments have demonstrated that certain lncRNAs can act as “sponges” for miRNAs by sharing common miRNA response elements (MREs), which further regulate the gene expression of the UPR pathway. This interaction leads to post-transcriptional regulation by inhibition of miRNAs [45]. In a study, it has been demonstrated that lncRNA OR3A4 has been related to the advancement of osteosarcoma (OS). Mechanistically, OR3A4 acts as a sponge for miRNA miR-1207-5p. The downregulation of OR3A4 led to an increased miR-1207-5p levels, suppressing its target enzyme glucose-6-phosphate dehydrogenase (G6PD) expression within these OS cells. As G-6-PD is pivotal in the pentose phosphate pathway (PPP), the diminution of OR3A4 caused decreased NADPH production, glucose consumption, and lactate generation. The decline in NADPH levels due to OR3A4 depletion augments the redox state, elevating ROS levels and triggering ER stress in OS cells [46].

**Fig. 5:**
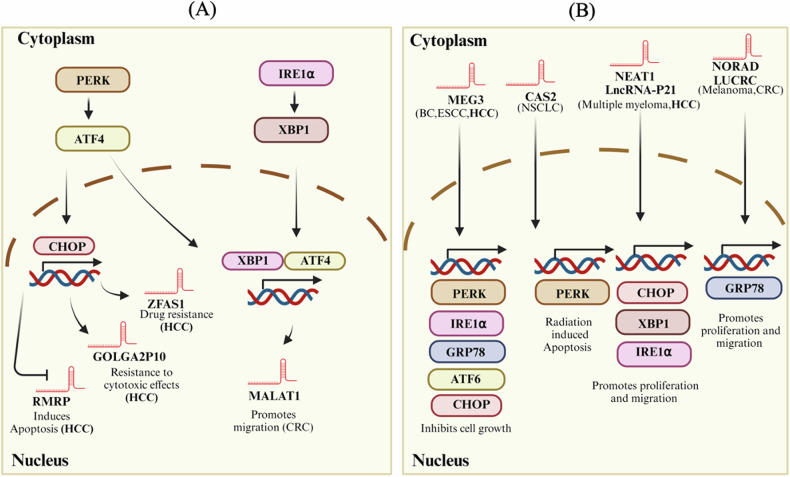
An intricate relationship between ER stress and lncRNAs: bidirectional relationship between ER stress and lncRNAs in the context of the UPR pathway. It highlights how UPR pathways regulate the expression of specific lncRNAs and how these lncRNAs, in turn, modulate key players in the UPR pathway, affecting various cellular processes. **A** The UPR pathway regulates the expression of lncRNAs, and (**B**) lncRNA modulates the key players of the UPR pathway. (Created with Biorender. com).

Figure 5 depicts the direct mechanisms by which ER stress and lncRNAs are interconnected. Despite their role in inducing apoptosis in various cancers, the mechanisms by which cancer cells evade apoptosis triggered by ER stress and the function of lncRNAs in mediating the same have not been thoroughly investigated.

### The interplay between ER stress and lncRNAs in hepatocellular carcinoma

ER stress, a cellular response to protein misfolding, is increasingly recognized as a pivotal factor in HCC pathogenesis. This complex cellular response is intricately linked to the dysregulation of lncRNAs. However, only a few studies have shown the mechanism of lncRNA regulating the induced ER stress in HCC. For instance, a study by Zhang et al. showed that lncRNA Gas 5 has a significant role in inhibiting tumor growth in HepG2 cells via the activation of the CHOP-dependent ER stress pathway [35]. Another study showed that MEG3 is downregulated in HCC and inhibits the proliferation along with the induction of apoptosis via the activation of the ER stress pathway. Also, activation of NF-ΚB is necessary, which activates the p53 in the context of ER stress [38]. In addition, MEG3 has been widely studied in the context of ER stress in various cancers like breast cancer, cervical carcinoma, and colorectal carcinoma and can act as the potential therapeutic target for the prevention of tumor progression [47–49]. LncRNA GOLGA2P10 plays a crucial role in safeguarding tumor cells against apoptosis. A significant increase in GOLGA2P10 was found in the HCC tissues and is related to the shorter recurrence-free survival in HCC patients. PERK/ATF4/CHOP activates GOLGA2P10 and modulates the activity of bcl-2, a regulator of apoptosis, and exerts a protective effect for the tumor cells and thus can act as the target for anticancer therapy [50]. In HCC cells, PERK downregulates lncRNA RMRP, leading to the induction of apoptosis and thus influencing the progression of HCC [51]. Recent studies have implicated the lncRNA ZFAS1 as a potential biomarker associated with sorafenib resistance. Sorafenib’s therapeutic effect is often counteracted by the PERK/ATF4 pathway activation, which paradoxically upregulates ZFAS1 expression [52]. In addition to this, another lncRNA NEAT1v1 (nuclear paraspeckle assembly transcript 1 variant 1) activates the AKT pathway via SOD2 leading to sorafenib and lenvatinib resistance with an increase in the sensitivity of HCC cell lines to capivasertib. This indicates the shift of growth modality from MEK/ERK-dependant to AKT- dependent via SOD2. Moreover, NEAT1v1 or SOD2 knockdown exacerbates ER stress, although AKT suppression occurs independently of ER stress [53]. NEAT1 is also upregulated in multiple myeloma and induces proliferation, migration, and invasion via increased CHOP, XBP-1, and IRE-1 expression. Liu et al. found that SNHG6 is found to be increased in HCC and is related to the poor prognosis and increased tumor progression [54]. LincRNA-p21 is another lncRNA that causes HCC growth inhibition and confers drug resistance by activating ER stress [55]. These findings suggest that ZFAS1 may serve as a prognostic indicator for predicting patient response to sorafenib and a potential therapeutic target to overcome drug resistance in HCC.

**Fig. 6:**
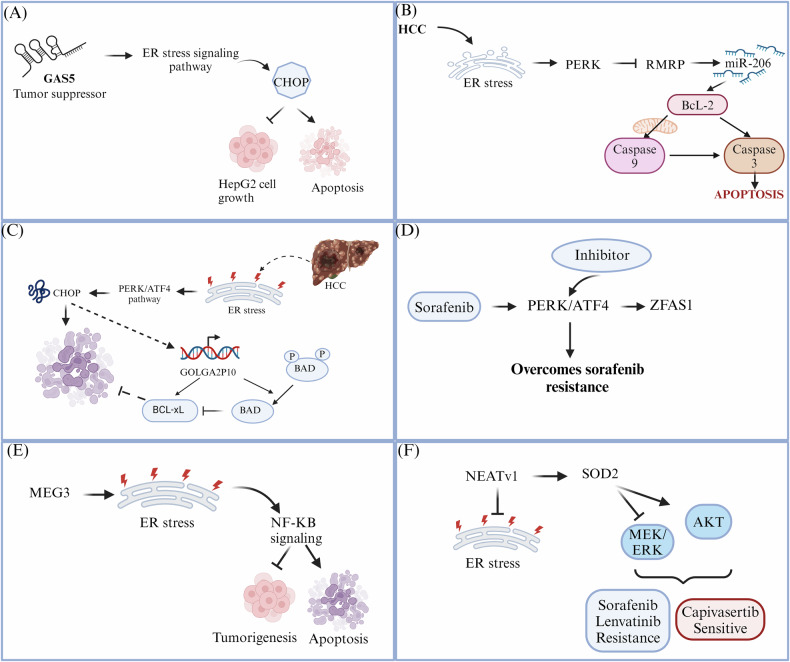
LncRNAs in ER stress-driven HCC progression. **A** GAS5 inhibits the proliferation and induces apoptosis of HepG2 cells in a CHOP- dependent pathway (**B**) PERK inhibits RMRP to induce apoptosis in HCC cells via BcL-2/caspase 3 pathway (**C**) PERK activates GOLGA2P10 in a CHOP-dependent manner to exert a protective effect for tumor cells via activating apoptosis. **D** Sorafenib causes activation of ZFAS1 via PERK/ATF4 pathway (**E**) MEG3 overexpression activates ER stress which further upregulates NF-KB signaling inducing apoptosis and impedes tumorigenesis (**F**) NEATv1 inhibits ERK pathway and activates AKT via SOD2 which confers sorafenib and Lenvatinib resistance with sensitivity to capivasertib. (Created with Biorender. com).

Figure 6 illustrates the intricate involvement of specific lncRNAs in the progression of HCC driven by ER stress. Altogether, these findings suggest that ER stress-related lncRNAs in different cancers can prove to be potential biomarkers for the diagnosis of HCC. Also, these can be targeted for the various potential therapeutic options for the treatment of HCC. However, all of these studies are in the in vitro and in vivo models of the HCC and further require the clinical validation on the larger cohort of HCC patients.

### Diagnostic and prognostic significance of lncRNAs in HCC

Biomarkers represent a promising frontier in fostering advancement in the disease diagnosis, treatment, and prevention, ultimately improving patient outcomes and overall understanding of complex diseases. The biomarkers can be detected earlier in various body fluids like tissue, serum, plasma, and urine and thus play a crucial role in identifying tumors at their early stage [56]. The discovery of biomarkers is facilitated by cutting-edge techniques that involve proteomics, transcriptomics, whole genome sequencing, and arrays [57, 58] Apart from serum and tissue biomarkers, miRNAs and lncRNAs are well- implicated as biomarkers in HCC. For example, some of the miRs that are differentially regulated in the HCC tissues in comparison to the healthy controls include miR-106, miR-122, miR-183, miR-21, miR-483, miR-15b (upregulated) and miR-92a, miR-16, miR-199a (downregulated) are considered as the diagnostic biomarkers in the HCC. However, miR-10b, miR-122, miR-124, miR-155, miR-22, and miR-221 are some of the miRs associated with poor prognosis and poor survival in HCC [59]. Not only miRNAs, lncRNAs also show notable dysregulation and can act as diagnostic and prognostic markers in HCC [58].

For example, higher expression of oncofetal mRNA H19 has been observed in the fetus. In fact, H19 is known to be higher in HCC cases than the expression of AFP [58]. Some more lncRNA that show potential prognostic and diagnostic values are described below:

1. GAS5-AS1 (Antisense RNA of growth arrest-specific transcript 5): GAS5, the sense RNA of GAS5-AS1, is known to be downregulated in HCC tissues, is related to the proliferation and invasion in HCC. Wang et al. reported that the expression levels of GAS5-AS1 were lower in HCC tissues, and this downregulation is linked with the degree of TNM stage, differentiation, and glucose levels in patients. The observations also revealed the association of decreased expression of GAS5-AS1 with poor prognosis and poor survival [60].
2. GAS5 (Growth arrest-specific transcript 5): Previous literature findings have reported a significant decrease in the expression of GAS5, which was further linked to advanced tumor progression. Moreover, GAS5 serves as an independent marker to predict the clinical outcome of HCC patients [61].
3. HOXB-AS1 (Hox cluster antisense RNA 1): HOXB-AS1 is significantly upregulated lncRNA in HCC, and acting as an oncogene it promotes the proliferation and metastasis of HCC cells. Apart from this, higher expression of HOXB-AS1 is also linked with the overall poor survival of HCC patients and thus holds great potential as a valuable biomarker for early diagnosis and prognosis in HCC patients [62].
4. MALAT1 (Metastasis-associated lung adenocarcinoma transcript 1): MALAT1 holds the potential to be used as a prognostic marker in individuals with HCC who have undergone liver resection as its expression is notably elevated in HCC and is linked to the unfavorable prognosis in HCC patients [63]. Moreover, Toraih et al. demonstrated that circulatory MALAT1 might act as the putative non-invasive prognostic and diagnostic biomarker showing a worse liver failure score in HCV- related HCC patients with traditional markers [64].
5. HOTAIR (HOX transcript antisense intergenic RNA): In HCC, HOTAIR has been identified as an oncogene playing a crucial role in promoting tumor growth and HCC development while suppressing apoptosis. Additionally, HOTAIR appears to influence HCC metastatic progression through the cell adhesion pathways. This shows the HOTAIR as a promising diagnostic marker in HCC [65].
6. TUG1 (Taurine upregulated gene 1): TUG1 lncRNA is already reported to be overexpressed in HCC patients. Lin et al. showed that TUG1 and AFP have a strong positive correlation, and both are related to poor survival in HCC patients, thus depicting the utility of TUG1 as the prognostic marker in HCC patients [66].
7. CASC9 (Cancer susceptibility candidate 9): A significant increase in the expression is seen in HCC patients compared to the normal samples. Analysis of CASC9 expression showed that the low expression of CASC9 is correlated with higher survival. In addition to this, pathological features like advanced TNM staging, AFP, lymph node metastasis, and tumor size were also positively linked with the overexpression CASC9 [67].
8. LRB1 (Light response BTB1): Increased LRB1 shows a significant association with the risk of HCC as its levels are positively correlated with the expression of AFP, tumor stage advancement, venous invasion, and poor recurrence-free survival rates. Therefore, the serum levels of LRB1 act as an early indicator for diagnosis and tumor prognosis in HCC patients [68].
9. SBF2-AS1 (Set binding factor 2 antisense RNA 1): Zhang et al. demonstrated that elevated SBF2-AS1 is associated with poor prognosis and acts as the novel biomarker in HCC [69].
10. LINC01224 (Long intergenic non-protein coding RNA): Literature findings report that LINC01224 gets elevated in liver cancer with a poor prognosis and is a potential biomarker ().
11. SNHG15 (Small nucleolar RNA host gene 15): In HCC, SNHG15 is a well-known oncogene that demonstrates significant overexpression and is also closely associated with histological grade, TNM staging, vein invasion, and poor overall survival leading to HCC development and progression [70, 71].
12. AK021443: Researchers have reported that lncRNA AK021443 exerts tumor-promoting effects in HCC and is known to be upregulated in the HCC tissues in comparison to corresponding non-cancerous tissues and is a powerful marker to predict HCC patients because of its association with lymph node metastasis, advanced TNM stage, and shorter overall survival [72].
13. PlncRNA-1: PlncRNA has been well implicated in the progression of HCC. HCC tissues exhibit elevated PlncRNA-1 expression, and this upregulation correlates significantly with advanced tumor stage, metastasis, and poor patient prognosis [73].
14. MKLN-1 (Muskelin 1): YAP-1 (Yes- associated protein-1) is a vital hypoxia response protein in HCC. YAP-1 can bind to HIF-1α and maintain its stability to activate the aerobic glycolysis, but a study by Guo et al. for the first time proved that MKLN-1 causes YAP-1 accumulation and hence tumor progression, thus can be used as an upstream factor in HCC for its diagnosis and prognosis [74].

**Fig. 7:**
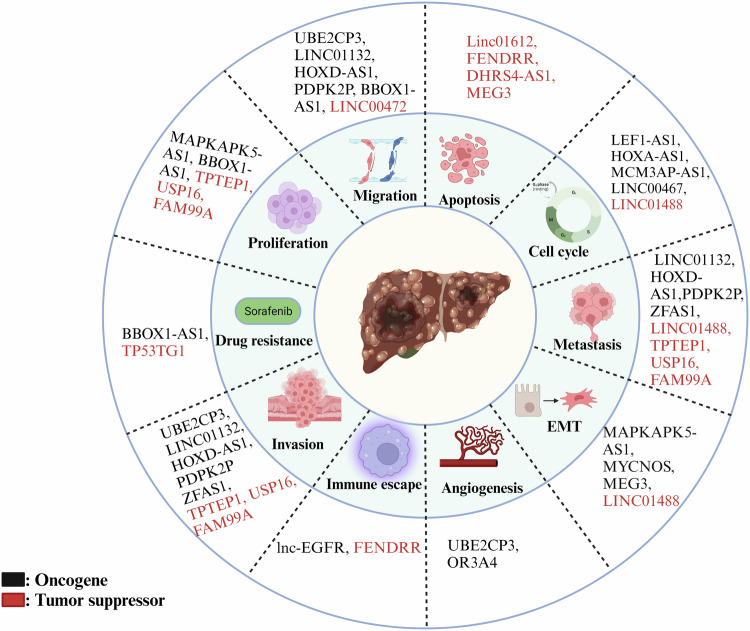
LncRNAs and their association with cancer hallmarks in HCC. (Created with Biorender. com).

All the above-discussed lncRNAs have been implicated in the diagnosis and prognosis of HCC because they correlated with the advanced tumor stage, metastasis, and a significant dysregulation in HCC tissues in comparison to the corresponding non-cancerous tissues of HCC patients (summarized in Fig. 7). GAS5 and GAS5-AS1 are the only two lncRNAs that are downregulated in the HCC tissues, whereas the other lncRNAs which include MALAT-1, HOXB-AS1, HOTAIR, TUG1, MKLN-1, etc. are the lncRNAs which are upregulated in HCC tissues when compared to normal tissues and their expression are related to poor prognosis and poor overall survival. Apart from all these, some other lncRNAs that might act as the prognostic or the diagnostic markers in HCC include lncRNA CSMD1-1, HOXA-AS2, UC001kfo, PTTG1 and PDIA3P1 [75–79]. The expression of lncRNA CSMD1-1 is increased in HCC and plays a crucial role in driving the progression of the disease by activating the Myc signaling pathway. It could serve as a novel prognostic marker and holds significant potential as a therapeutic target for HCC [76]. LncRNA HOXA-AS2 exhibited abnormal upregulation and appears to play a significant role in cancer stemness, which is associated with an unfavorable prognosis [77]. Moreover, literature reports that lncRNA UC001kfo notably promotes HCC proliferation and metastasis by targeting α-SMA [78]. PTTG3P activates the PI3K/AKT signaling pathway in HCC and plays a crucial role in tumor growth and metastasis by upregulating PTTG1 which can be a potential target for gene-based therapy [79]. PDIA3P1 promotes migration, invasion, and proliferation while concurrently reducing apoptosis of HCC cells [75]. Altogether, all of these lncRNAs have a role in the progression of HCC by promoting migration, invasion, and the stemness of HCC and might act as the potential diagnostic or the prognostic marker and can even be used as the therapeutic target in gene-based therapy to increase the overall survival.

## Prospective application of lncRNAs in HCC: a potential catalyst waiting to emerge or an underdeveloped structure in the sand?

Inspite of considerable strides that have been made in the advancement of therapeutic interventions for HCC, the overall prognosis is still bleak, which is majorly attributed to challenges like late-stage diagnosis, chemotherapy failure, instances of recurrence, and the absence of robust molecular targets for the effective treatment. Despite all this knowledge, the multifaceted nature of HCC has made it difficult to find a potential target for the disease.

While existing research demonstrates the effectiveness of sequence-specific siRNAs for lncRNA targeting, a gap exists in applying knockout models for this purpose. Previous literature shows the usage of specific base pairing principles to target the lncRNAs with some small molecules like siRNAs. Various methods for the knocking out of lncRNAs have been proposed, including the complete deletion of the respective gene, removal of lncRNA promoters, and the incorporation of premature polyadenylation cassette [80]. Some of the approaches used to target the lncRNAs and modulate their expression include the usage of siRNAs, aptamers, natural antisense transcripts (NATs), antisense oligonucleotides (ASO), locked nucleic acid (LNA) Gapmers, ribozymes, small molecule inhibitors, and RNA destabilizing elements (RDEs) and CRISPR-Cas9 based strategies [81]. For example, the study showed that the injection of ASOs targeting lncRNA MALAT1 mitigated tumor propagation in nude mice [82]. Similarly, the growth of HCC xenografts was inhibited by using cholesterol-conjugated siRNA, which targeted lnc-UCID [83]. However, the major challenge in these approaches is achieving the target- specific delivery. Furthermore, siRNAs get sequestered into endosomes, which means they do not reach the target RNA in the cytoplasm/nucleus [84]. Apart from this, lncRNAs have lower sequence similarity across species; thus, translating data from animal models into humans poses a substantial challenge. Still, some of the studies done in the context of HCC have proved the use of the lncRNAs as an effective treatment perspective. For instance, MEG3 coated with nanoparticles has been shown to cause a significant reduction in the expression of tumor markers and decreased expression of hepatic PCNA [85]. A precedent exists for applying FDA-approved nanoparticles as nucleic acid delivery vehicles due to their enhanced permeability characteristics. This strengthens the rationale for utilizing these nanoparticles as novel and efficacious delivery systems for lncRNAs within the context of lncRNA-targeted therapeutic strategies [86]. Chen et al. depicted that the increased efficacy of the drug Lenvatinib, which is an FDA- approved drug for the HCC treatment was found with the knockdown of MKLN1 due to the increased apoptosis in the HCC [87]. On the other hand, LINC00082 (activated by ATF2) is known to have an oncogenic role by sponging of miR-214-3p, which further activates centrosome protein M (CENPM), which can be a potential target in the treatment of HCC [88]. Apart from this, the advent of CRISPR/Cas9 technology has revolutionized the exploration of the non-coding genome, enabling precise and high-throughput investigation of its genetic and functional properties. Recent advances suggest the usage of CRISPR-ko to directly or indirectly knockout the lncRNA, CRISPRi (CRISPR interference) for the knockdown of lncRNAs and CRISPRa (CRISPR activation) for the overexpression of lncRNAs. In a carefully designed proof-of-concept animal study, researchers found that administering a CRISPR/Cas9 system targeting GMAN significantly reduced gastric cancer cell metastasis and improved overall survival in mice [89]. While off-target cleavage events can occur in practical applications, CRISPR/Cas9 is a highly flexible and selective genome editor, making it an exceptional tool for genetic modifications. To minimize the off-target effects and increase the repression efficiency of CRISPR, dCas9 can be combined with the transcriptional repressor domain like the kruppel-associated box (KRAB) domain [90]. Also, a study by Liu et al. [91] suggests the CRISPR-mediated knockdown of MEG3 caused the decrease in the proliferative capacity in the xenograft model of mice [92]. Unlike CRISPRi, CRISPRa enhances lncRNA expression by recruiting transcriptional activators to the lncRNA promoter region. Like CRISPRi, CRISPRa employs a dCas9 protein, but instead of a repressor, it is fused to a transcriptional activator domain, such as the VP64 domain [93]. Beyond modulating the expression of lncRNAs using CRISPRi and CRISPRa, the CRISPR-Cas9 system can be employed for the creation of complete knockouts which involves using Cas9 and a specific sgRNA to introduce double-strand breaks (DSBs) at the lncRNA genomic locus [94]. For instance, MALAT1 knockout cells were created using CRISPR-Cas9 technology, which revealed the role of MALAT1 in the regulation of alternative splicing and its contribution to lung cancer metastasis [95]. Altogether, this suggests the potential of CRISPR in modulating lncRNAs. However, while the promise of this technology is immense, it remains in its early stages, with numerous technical and ethical challenges, which include the off-target effects, delivery methods, and regulatory capacities, that must be addressed before it can be broadly implemented for cancer prevention. Foremost, inadvertent binding and cleavage of genomic DNA at sites other than the intended target site can lead to off-target effects [96]. Secondly, viral particles, nanoparticles, and liposomes have been used as the delivery methods that can lead to the impediments like toxicity and immunological reactions which need to be addressed [97]. Also, it is important to consider the localization of lncRNAs, interaction with other molecules and context-specific expression before designing the CRISPR- mediated modulation of lncRNAs [98].

The foremost thing is that the intricacies of HCC regulation are more obscure than anticipated because of the diverse range of lncRNAs to be associated with the disease. Moreover, deeper exploration is required to deal with the potential fluctuations in the expression of lncRNAs throughout the disease progression. Apart from this, selecting the specific lncRNA for targeting could be contingent upon the individualized expression patterns observed in each patient.

Another critical consideration as the major challenge in context to the lncRNAs as a potential target is the intricate interplay among the lncRNAs and various other ncRNAs [99]. For instance, several oncogenic miRNAs are sequestered by both lncRNAs and circRNAs, which implies that the concurrent targeting of the silencing of both will be a necessary approach [100]. Moreover, the repercussions of targeting the lncRNAs that are not upregulated uniformly in HCC remain unclear.

### Conclusive remarks and future perspectives

Hepatocellular carcinoma is a significant contributor to cancer-related deaths worldwide. The incidence and mortality rates are on the rise, accompanied by a decrease in the survival rate. In light of this alarming scenario, it is crucial to identify effective strategies or targets capable of suppressing the initiation, progression, metastasis, and invasion of HCC. The existing evidence in the literature shows that the tumor cells have an increased demand for the synthesis of folded proteins, an imbalance in the synthesis, and the degradation of misfolded proteins activate the UPR in reaction to these alterations and unfavorable environmental conditions. In this context, UPR acts as a mechanism to promote oncogenesis, contributing to various facets of cancer development. Due to its ability to activate both pro-survival and pro-apoptotic signals, therapeutic strategies for tumors can leverage agents that either induce or inhibit key molecules involved in UPR. For instance, PERK inhibitors like GSK260641 and ATF6 (exemplified by 16F16), along with the CHOP inducer (like DK143), present promising avenues for intervention in cancer treatment [101–103]. Even though there has been success in preclinical cancer models by inhibiting the major players of UPR with small molecules, systemic targeting of UPR factors may lead to unintended long-term side effects for patients [104]. In addition, as a response to the increased ER stress, tumor cells undergo a sequence of biological alterations aimed at adapting to their growth conditions, including regulating non-coding RNA expression. On the other hand, non-coding RNAs can also regulate the UPR components. Thus, UPR-related genes can act as both the upstream regulators and the downstream effectors of ncRNAs, forming the crosstalk between the two [16]. This review highlighted the complex interconnected network of lncRNA and UPR, which can be targeted together for better therapeutic interventions Accumulating evidence over the years supports the notion that non-coding RNAs can serve as viable therapeutic targets in oncology [105]. For example, miRNAs are also commonly categorized as tumor suppressors or oncogenes, considering the specific cell-type and other factors. Similarly, in the context of ER stress, miR-199A-5p demonstrates a cytoprotective effect when IRE1α is inhibited and induces apoptosis when GRP78 or ATF6 is inhibited [106]. Consequently, interventions directed at the lncRNA-miRNA-UPR pathway emerge as the crucial strategy for cancer therapy, with numerous non-coding RNAs demonstrating the mechanism of action within this framework as lncRNAs majorly act by sponging the miRs [107]. Investigating and targeting the upstream regulatory molecules influencing the miRNAs-mRNA network will be more beneficial. Moreover, given the association of lncRNAs with the sensitivity of HCC to radiotherapy and chemotherapy, they can serve as molecular markers predicting the efficacy of therapeutic interventions. Furthermore, they can also be utilized as targets combined with chemotherapy or radiotherapy to enhance the sensitivity of the treatment. In addition, intricate networks formed by lncRNA and miRNAs play a crucial role in regulating tumor microenvironment (TME) by influencing EMT, hypoxia response, glycolysis, and tumor evasion. Within TME, hypoxia fosters glycolysis, EMT, and the instauration of an immunosuppressive milieu. Concurrently, certain promoter lncRNAs contribute to positive signaling loops that intensify hypoxia, trigger M2 polarization of macrophages, fostering EMT, and thus exacerbating immune evasion.

Currently, the majority of investigations are in the early stages of basic research with a limited presentation of clinical applications of lncRNAs. As a result, significant advancements in cancer treatment are anticipated through targeted modulation of lncRNAs and ER stress in the foreseeable future.
